# Quantification of the Kynurenine Biomarker in Saliva Using Smatphone-Based Fluorescence Digital Imaging

**DOI:** 10.1021/acsomega.5c11977

**Published:** 2026-06-15

**Authors:** José Gouveia da Silva Neto, Luís Vinícius Gonçalves de Melo, José Ailton Mota Nascimento, Helayne S. de Sousa, João Paulo de Almeida, Severino Carlos Oliveira, Vagner Bezerra dos Santos

**Affiliations:** † Department of Fundamental Chemistry, 28116 Federal University of Pernambuco, Av. Jornalista Anibal Fernandes, S/N, Cidade Universitária, Recife, Pernambuco 50740-560, Brazil; ‡ Department of Chemistry, 67744 Federal Rural University of Pernambuco, Dom Manuel de Medeiros Street, S/N, Dois Irmãos, Recife, Pernambuco 52171-900, Brazil

## Abstract

L-tryptophan (Trp) is an essential amino acid whose metabolism produces biologically relevant compounds such as serotonin, melatonin, kynurenine (Kyn), kynurenic acid (Kyna), and nicotinamide adenine dinucleotide (NAD+). Alterations in kynurenine pathway metabolism have been associated with several neurological and neurodegenerative disorders. Due to this importance, this study presents a fluorescence digital image-based (FDIB) analytical method for the quantification of kynurenine (Kyn) using a smartphone. The method is based on the derivatization reaction of Kyn with dansyl chloride (DNS-Cl), since the Kyn-DNS product exhibits high fluorescence when excited at 365 ± 50 nm. Thus, a UV-LED chamber was fabricated on a 3D printer, and it was used for excitation, as well as for capturing the fluorescence of Kyn-DNS using a smartphone, leading to the Kyn-DNS/3Dchamber/FDIB analytical method. The proposed method showed a linear range from 0.50 to 7.00 μmol L^–1^, limit of detection (LOD) of 0.05 μmol L^–1^ and precision below 2.68%, making it suitable for quantifying Kyn in biological samples such as saliva and urine. The accuracy of the method was evaluated by recovery tests, and a recovery rate from 92.1% to 106% was achieved for synthetic saliva samples. A good agreement at a 95% confidence level with n = 3 was obtained when comparing the FDIB results with those supplied by spectrofluorometry and HPLC-UV methods, which certify the accuracy of the method. The Kyn-DNS/3Dchamber/FDIB method proposed reduces several analytical steps normally required in conventional approaches, such as removal of excess derivatizing agent, purification, solvent evaporation, analyte preconcentration, and filtration. This simplification makes the Kyn-DNS/3Dchamber/FDIB method faster, more cost-effective, less waste-generating, and environmentally friendlier compared to spectrofluorometry and HPLC methods.

## Graphical abstract

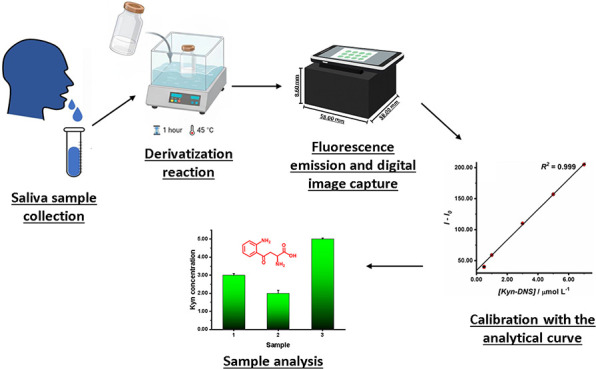

## 1 Introduction

The kynurenine pathway (KynP) is the major route of the essential amino acid tryptophan (Trp) degradation, in human metabolism for the generation of different bioactive molecules, such as kynurenine (Kyn), kynurenic acid (Kyna), quinaldic acid (Quina), picolinic acid (Pic), quinolinic acid (Quin), and others.^1,2^ Trp also can be metabolized via the serotonin pathway (SerP) for the production of serotonin and melatonin, both of which are important molecules for the organism.^1,2^ Kyn and other metabolites from the KynP are bioactives and can interact with different species and biochemical processes.^1–3^ Kyn can act as an inhibitor of T cells, decreasing the T helper 1 (Th1) and increasing the T helper 2 (Th2) responses, thereby affecting the immune system^3,4^ and also interacts with anti-inflammatory molecules such as Human Lymphocyte Antigen-G.^3–6^ According to Bizjak et al., the concentration levels of Kyn in biofluid samples from a normal group of humans, including blood bank donors, are 2.79 ± 0.61 μmol L^–1^ (n = 302) in serum samples and 0.50 to 0.70 μmol L^–1^ (n = 117) in saliva samples.^7^ Bizjak et al. also showed that in patients with covid-19, the levels of Kyn in human serum samples increase to 10.81 ± 8.80 μmol L^–1^ (n = 85).^7^ Fuchs et al. demonstrated that the levels of Kyn in patients with human immunodeficiency virus type 1 infections (HIV-I seropositives) are 3.45 ± 0.14 μmol L^–1^ (n = 42), while HIV-I seronegative control patients presented levels of 2.31 ± 0.23 μmol L^–1^ (n = 11).^8^ From this perspective, Kyn detection and quantification have been investigated as a biomarker for the early diagnosis and treatment of neurodegenerative and inflammatory human diseases.^1–11^

Different analytical methods have been proposed for Kyn’s detection and quantification in biological fluids, most of them using analytical techniques as chromatographic methods, such as high-performance liquid chromatography (HPLC) with UV,^12^ fluorescence,^12–14^ mass spectrometry (MS),^15–17^ and electrochemistry detection using differential pulse voltammetry (DPV)^16–20^ However, alternative methods based on digital imaging using smartphones have not been reported in the literature for Kyn quantification.^21,22^

The digital image-based (DIB) method is an analytical approach that uses digital cameras, webcams, or smartphones to quantify the color or fluorescence intensity of chemical reactions, correlating the numerical values extracted from the images with the concentration of the analyte.^21–23^ It stands out as a fast, practical, and low-cost alternative to spectrofluorometric methods, combining advantages such as portability, operational simplicity, low energy consumption, and the wide availability of imaging capture devices.^23,24^ These characteristics allow the creation of customized analytical systems, often combined with 3D-printed structures, resulting in compact and versatile methods.^21^ DIB has wide applicability on site analyses, especially in locations with limited resources, and has been used successfully in food,^25,26^ beverages,^21^ fuels,^27,28^ and environmental samples.^29,30^ Its reliability depends on image acquisition conditions, color model selection, and data processing.^31^ Excessive brightness, shadows, distance, and focus can also compromise results.^31^ Thus, standardization of image capture conditions is essential, and it is common to use dark chambers to isolate the sample from external interference and ensure reproducibility by reducing spurious signals and maintaining consistent distance and focus during image acquisition.^21,26,32^

Digital image processing involves the conversion of digital images into useful numerical data using appropriate color systems.^23^ Among the different models, HSV (Hue, Saturation, and Value), CIELab (Commission Internationale de l’Éclairage, L scale, light vs. dark, a scale, red vs. green, and b scale, yellow vs. blue), and RGB (Red, Green, and Blue) have been used. However, the RGB model is the most widely used due to its simplicity and compatibility with digital camera sensors and smartphones.^23,28,33,34^ In addition to the additive models mentioned, the subtractive CMY (Cyan, Magenta, and Yellow) and CMYK (Cyan, Magenta, Yellow, and Key, black) models are also used.^23,28,34^ These models can be used in software such as ImageJ for personal computers^23^ or applications such as ColorGrab, Photometrix, and ColorLab for smartphones. These softwares and apps allow for the quick and easy acquisition of the average and standard deviation of color channel intensities under analysis.^35–38^ The analytical response (AR) is often expressed as the difference between the intensity of the sample and that of an analytical blank.^22,39^ Alternatively, AR can be obtained by vector operations, such as the sum of the RGB channels or the calculation of the Euclidean distance, the latter being especially useful because it does not require the prior selection of a dominant channel, uses the entire visible spectrum, and proves to be more robust in complex matrices.^21,39^

The fluorescence digital image-based method (FDIB) represents an extension of the conventional DIB, incorporating fluorescence detection as the analytical mechanism.^38–41^ Unlike colorimetry, which relies on the partial absorption of visible light, fluorescence involves the emission of radiation by a chemical species, assisted by an excitation source, commonly, an ultraviolet radiation.^38–41^ Capturing this emission through digital devices enables the quantification of analytes with high sensitivity and selectivity, making the method particularly useful for complex samples or those containing low concentrations of target chemical species.^41^ The experimental setup of an FDIB system may vary depending on the application, but it generally includes an excitation radiation source (such as light emission diodes -LEDs), a dark chamber adapted to block ambient light and minimize optical noise, and a digital device for image capture, such as a smartphone, webcam, or conventional camera.^21,43^ FDIB stands out for its high signal-to-noise ratio and capability to detect analytes at low concentration levels^41^ with high portability.^21,42,44^

Kynurenine (Kyn) is an amino acid, and its fluorescence is weak; thus, a derivatization reaction is required to obtain high-sensitivity and high-selectivity methods.^45,46^ The derivatization reaction modifies Kyn differently from other biomarkers in that it reduces interferences.^45,47–50^ For amines, it is common to find different selective species for their derivatization, such as dansyl chloride (DNS-Cl).^26,45,51^ In the derivatization reaction between DNS-Cl and primary amines, the amino group acts as a nucleophile, attacking the sulfur of the sulfonyl group of DNS-Cl, which acts as an electrophile.^26,45^ Sometimes the reaction is performed in an alkaline medium, and the HCl produced is partially reacted to produce water^26,45,46,51^ (Figure 1).

**Figure 1:**
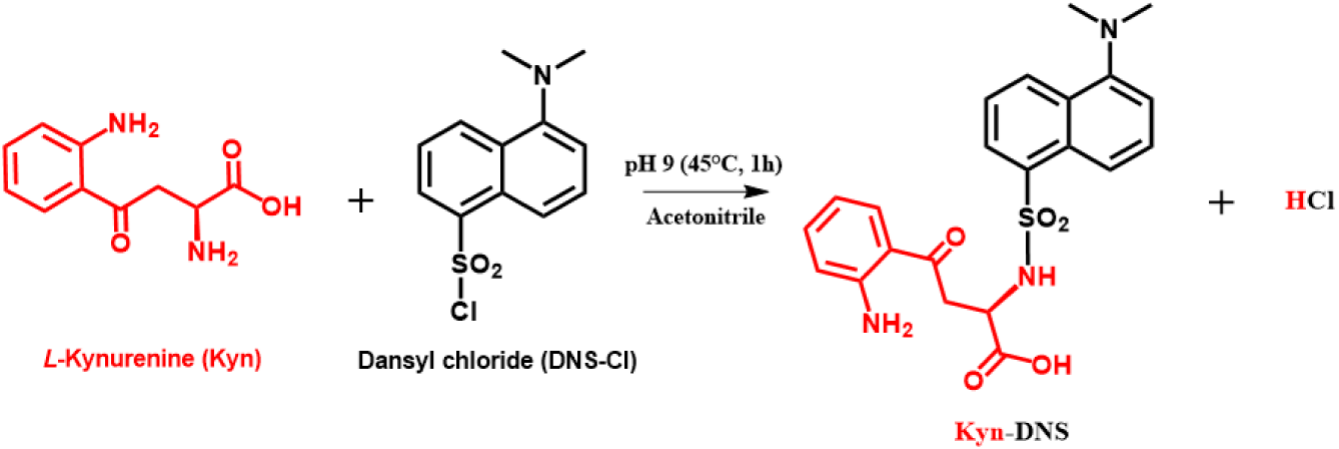
Reaction of Kyn with DNS-Cl to form the Kyn-DNS product.

Dai et al. performed the derivatization of Kyn with DNS-Cl for its determination in rat plasma samples using ultrahigh-performance liquid chromatography coupled with triple quadrupole mass spectrometry.^52^ They studied the reaction time (40 to 90 min), temperature (30 to 90 °C), pH (8.53 to 11.52), and DNS-Cl concentration (2 to 7 mg/mL). After balancing the stability and yield of derivatives, the optimized condition was 7 mg/mL DNS-Cl with a buffer pH of 9.78, kept at 80 °C for 70 min, allowing for stable and reproducible derivatization.^52^ The formation of Kyn-DNS adducts was measured by mass spectrometry in positive mode, where the precursor ion was detected at *m*/*z* = 442.4 and the fragment ion at *m*/*z* = 170.1.^52^ According to these and other authors, the derivatization reaction using DNS-Cl and its selectivity are influenced by reaction time, temperature, pH of the medium, and DNS-Cl concentration.^26,52–54^ Indeed, the longer the time, the greater the reaction yield; increasing the temperature promotes an increase in the reaction rate, while the pH depends on the target analyte, since chemical species can have different p*K*_a_ values in their structures, allowing for increased selectivity with control of the reactional pH.^26,52–54^

Yue et al. used DNS-Cl to dansylate biogenic amines in fresh meat samples using a microwave synthesizer.^53^ In this study, the following parameters were evaluated: reaction time (1 to 5 min), temperature (30 to 60 °C), and spinning speed (300 and 600 rpm).^53^ The derivatization reaction of the amines was evaluated using reversed-phase HPLC with a diode array detector (HPLC-DAD) for the determination of biogenic amines in chicken, beef, and mutton sold in the wet market.^53^ The results demonstrated that derivatization with DNS-Cl under optimized conditions, i.e, temperature and time of derivatization at 40 °C for 1 min, and a spinning speed of 600 rpm, allowed the determination of tryptamine, histidine, tyramine, and putrescine in the samples studied.^53^ Liu et al. described a method for the derivatization of 20 protein amino acids using DNS-Cl at pH 9.8, and their quantification in biological samples using liquid chromatography–mass spectrometry (LC-MS).^54^ Santos et al.^26^ reported, for the first time in the literature, the derivatization of biogenic amines using DNS-Cl with a fluorescence digital image-based (FDIB) method. The optimized derivatization presented a pH of 11, a temperature of 40 °C, and a time of 3 h. On the other hand, there are no reported analytical methods based on FDIB for the detection of Kyn.

From this perspective, this work aims for the first time to develop a new method for the detection and quantification of the biomarker Kyn in synthetic saliva samples using the FDIB technique, based on the derivatization of Kyn with DNS-Cl. For this, a chamber with UV-LEDs suitable for excitation and fluorescence image capture of the Kyn-DNS using a smartphone was developed (Kyn-DNS/3Dchamber/FDIB). Furthermore, the Kyn-DNS/3Dchamber/FDIB method was demonstrated to be simple, low-cost, and sensitive, requiring no additional analytical steps such as removing excess derivatizing agent, purifying, removing solvent, or preconcentrating the analyte, which are analytical procedures typically required in chromatographic, colorimetric, fluorometric, and other methods described in the literature.^45,47–50^ Thus, the present study was focused on the development of a method for the analysis of saliva samples. The recovery tests were performed in synthetic saliva with complex compounds, and no potential interferents were obtained. Moreover, the weak interference observed for citrate and other potential interferences on the Kyn-DNS method was less than 10%, and considering the micromolar concentration of Kyn, this result was acceptable. Moreover, the method presented a green metric of 79%, which contributes to green analytical purposes.

## 2 Materials and Methods

### 2.1 Reagents and Solutions

All reagents used for the experiments were of analytical grade and used as received. All the aqueous solutions were prepared using deionized water (resistivity >18.0 MΩ·cm) from a Millipore Milli-Q system (Merck, Saint Louis, USA). Kynurenine (Kyn), kynurenic acid (Kyna), uric acid (Ura), quinaldic acid (Quina), picolinic acid (Pic), sodium citrate, and dansyl chloride (DNS-Cl) were purchased from Sigma-Aldrich (São Paulo, Brazil). Sodium bicarbonate, sodium hydroxide, sodium dihydrogen phosphate, and sodium hydrogen phosphate were obtained from Neon (São Paulo, Brazil). Acetonitrile was purchased from Dinãmica (São Paulo, Brazil). Phosphate buffer solutions (PBS) (0.20 mol·L^–1^ sodium dihydrogen phosphate +0.20 mol·L^–1^ disodium hydrogen phosphate) were prepared (pH = 7.15 and pH = 10.00) with deionized water. The pH of the solutions was regulated with a 0.50 mol·L^–1^ NaOH solution. A stock solution of 1.20 mmol·L^–1^ Kyn was prepared in phosphate buffer at pH = 7.15. A stock solution of 2.40 mmol·L^–1^ DNS-Cl was prepared in acetonitrile. Stock solutions of 0.12 mol·L^–1^ Quina, Pic, and sodium citrate were prepared in deionized water. Stock solutions of 0.12 mol·L^–1^ Kyna and Ura were prepared in deionized water, and 150.00 μL of 0.50 mol·L^–1^ NaOH was added to achieve a final volume of 10.00 mL of solution.

### 2.2 Preparation of Saliva Samples

Saliva is an easily accessible biological fluid that can provide painless analysis and diagnosis for the patient compared to blood samples. It has already been demonstrated that Kyn and changes in its concentration can be detected in saliva samples.^7^ Consequently, analytical parameters, such as accuracy, precision, sensitivity, and selectivity of the proposed method were evaluated in synthetic saliva samples.^57–60^ The synthetic saliva samples were prepared following the protocol described by Rasheed et al. and Freire et al.,^42,60^ by mixing 1.00 g L^–1^ beef extract (HiMedia Laboratories Pvt. Ltd., Mumbai, India), 2.00 g L^–1^ yeast extract (HiMedia), 5.00 g L^–1^ protease peptone (HiMedia), 2.50 g L^–1^ hog gastric mucin type III (Sigma–Aldrich, St. Louis, MO), 0.20 g L^–1^ NaCl (Sigma–Aldrich, St. Louis, MO), 0.20 g L^–1^ KCl (Synth, Diadema, SP, Brazil), 0.30 g L^–1^ CaCl_2_ (Sigma–Aldrich, St. Louis, MO), 1.25 mL L^–1^ of a 40.00% (v/v) urea solution (Sigma–Aldrich, St. Louis, MO), and 1.00 L of deionized water.

### 2.3 Derivatization Reaction Method

Fluorescence derivatization reaction of Kyn was carried out with a fluorophore species, DNS-Cl, to produce a derivatized product, Kyn-DNS, following an adapted protocol described by Santos et al.^26^ In an amber vial, 2.00 mL of Kyn solution (1.20 mmol L^–1^) and 1.00 mL of acetonitrile were added, and the pH was adjusted to the range of 9.00 to 10.00 using a 1.00 mol L^–1^ NaOH solution. Then, 1.00 mL of DNS-Cl solution (2.40 mmol L^–1^) was added. The mixture was subjected to a derivatization reaction in a water bath at 45.00 °C for 1 h. In order to carry out the derivatization reactions, a magnetic stirrer with a 752A hot plate from Fisatom (São Paulo, Brazil) was used. Micropipettes from DelTECH (India) were employed to transfer volumes from 10.00 to 100.00 μL and 100.00 to 1000.00 μL. The saliva samples were derivatized following the same protocol for Kyn derivatization. In an amber vial, 2.00 mL of saliva and 1.00 mL of acetonitrile were added, the pH was adjusted to the range of 9.00 to 10.00 using a 1.00 mol L^–1^ NaOH solution, 1.00 mL of DNS-Cl solution (2.40 mmol L^–1^) was added, and finally, the mixture was subjected to a derivatization reaction in a water bath at 45.00 °C for 1 h.

### 2.4 Spectrofluorometry and High-Performance Liquid Chromatography Methods

Fluorescence measurements were carried out on a Hitachi F-7100 fluorescence spectrophotometer. The excitation wavelength was fixed at 380.0 nm, and the emission spectra were recorded in the range of 400.0–800.0 nm. Both excitation and emission monochromator slit widths were set to 5.00 nm. The photomultiplier tube voltage was adjusted to 400.00 V to optimize detector sensitivity. The scan speed was set to 1200.00 nm/min, and the response time was configured to 0.50 s.

The HPLC-UV method employed a Shimadzu HPLC LC-20A chromatograph with a diode array detector. The Kyn was analyzed at 360 nm without derivatization as reported by Parráková et al.^55^ For this, a ULTISIL column, AQ-C18, 250 × 4.6 mm, 5.0 μm, 120 A was used. A sample plug of 20 μL of sample volume was injected, and an isocratic elution using 15 mmol^–1^ potassium dihydrogen phosphate buffer solution (pH= 4.6) in methanol was used as the mobile phase at a flow rate of 1.0 mL min^–1^ for 10 min at 25 °C. Kyn was detected at a wavelength of 360 nm from a linear range from 0.5 to 7.0 μmolL^–1^. Before injecting the saliva skipped with Kyn (0.5 and 1.0 μmolL^–1^), the samples were pretreated according to Polson et al.^56^ However, proteins were precipitated by adding 0.1 mol L ^–1^ HCl and further centrifuged 10000 g for 10 min. This sample treatment is widely reported in the literature.^57–61^ The supernatant was diluted 1:1 (v/v) using the mobile phase, with the pH adjusted to 4.6 using PBS before injection into the chromatograph.

### 2.5 Fluorescence Digital Image-Based Method

A black chamber made of polylactic acid (PLA) was fabricated using a 3D printer (Creality Ender 3 Pro), with dimensions of 58.00 × 58.00 × 8.60 mm, containing two UV-LEDs emitting radiation at a maximum wavelength of 365 ± 50 nm, aimed at controlling the radiation applied to the samples. This wavelength band is similar to those used for excitation in the spectrofluorometer. For this, the chamber was powered using a stable 12 V power supply capable of supplying 1A for each UV-LED. The chamber is completely closed, and it is expected to obtain low spurious signals. Additionally, a 3D-printed polyethylene terephthalate glycol (PETG) microplate with 25 wells was employed. Each of the 25 wells has a capacity of 150.00 μL, which is useful for reducing waste generation, focusing on a greener analytical method. Image acquisition was performed using a Samsung Galaxy A35 smartphone with a 12-megapixel camera (Figure 2).

**Figure 2:**
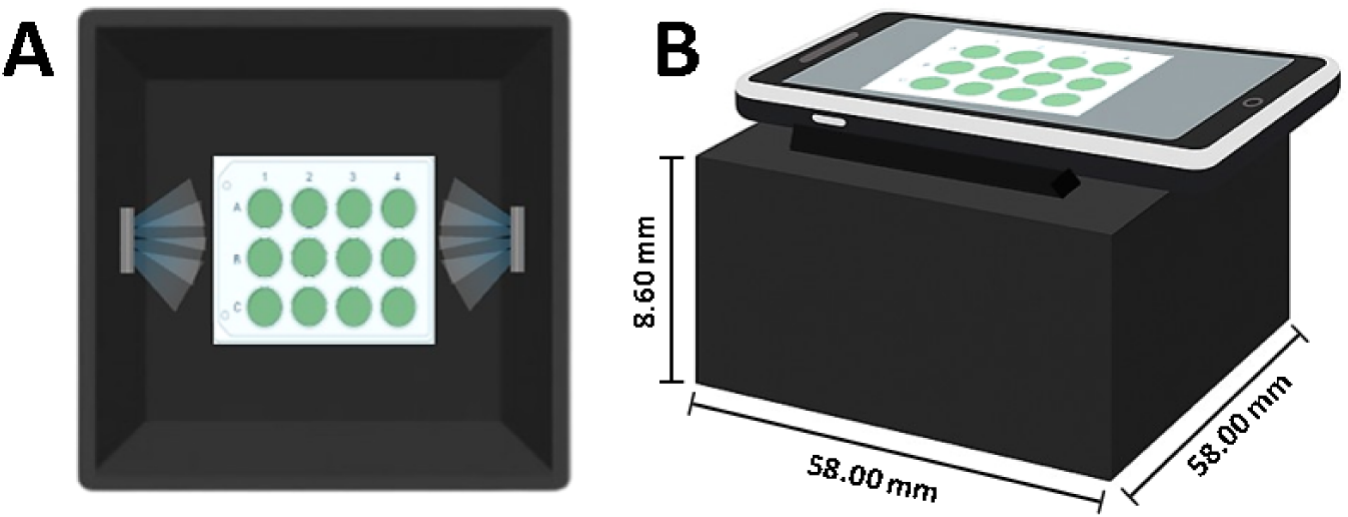
Kyn fluorescence analysis chamber developed for the proposed Kyn-DNS/3Dchamber/FDIB method: (A) upper internal view and (B) external view.

The procedure for quantifying Kyn was performed in three steps, Figure 3. Step 1 consisted of the derivatization reaction of Kyn with DNS-Cl to form the Kyn-DNS adduct. In step 2, after derivatization, the samples were diluted in a 1:10 ratio with acetonitrile, and a volume of 150.00 μL was transferred to the plate, which was then placed in the chamber. The fluorescence emission was measured using the smartphone 30 s after the 1 h reaction time, as described in step 3 (Figure 3). The images were analyzed using the ImageJ software and using the Color Grab app. Regions of interest (ROI), 300 × 410 pixels were selected, and the intensity values of the red (R), green (G), and blue (B) channels were extracted. Quantification was performed by subtracting the blank intensity (R_0_, G_0_, B_0_), which consists of DNS-Cl with or without the saliva sample (*I*_0_) from the sample intensity (*I*), which consists of Kyn-DNS or Kyn-DNS in the saliva sample, using the equation *I* – *I*_0_. Additionally, a vector was applied to account for the combined contribution of all three-color channels (RGB), eq 1

1 $$\mathrm{Vector}=\sqrt{{\left(R-R_{0}\right)}^{2}+{\left(G-G_{0}\right)}^{2}+{\left(B-B_{0}\right)}^{2}}$$

**Figure 3:**
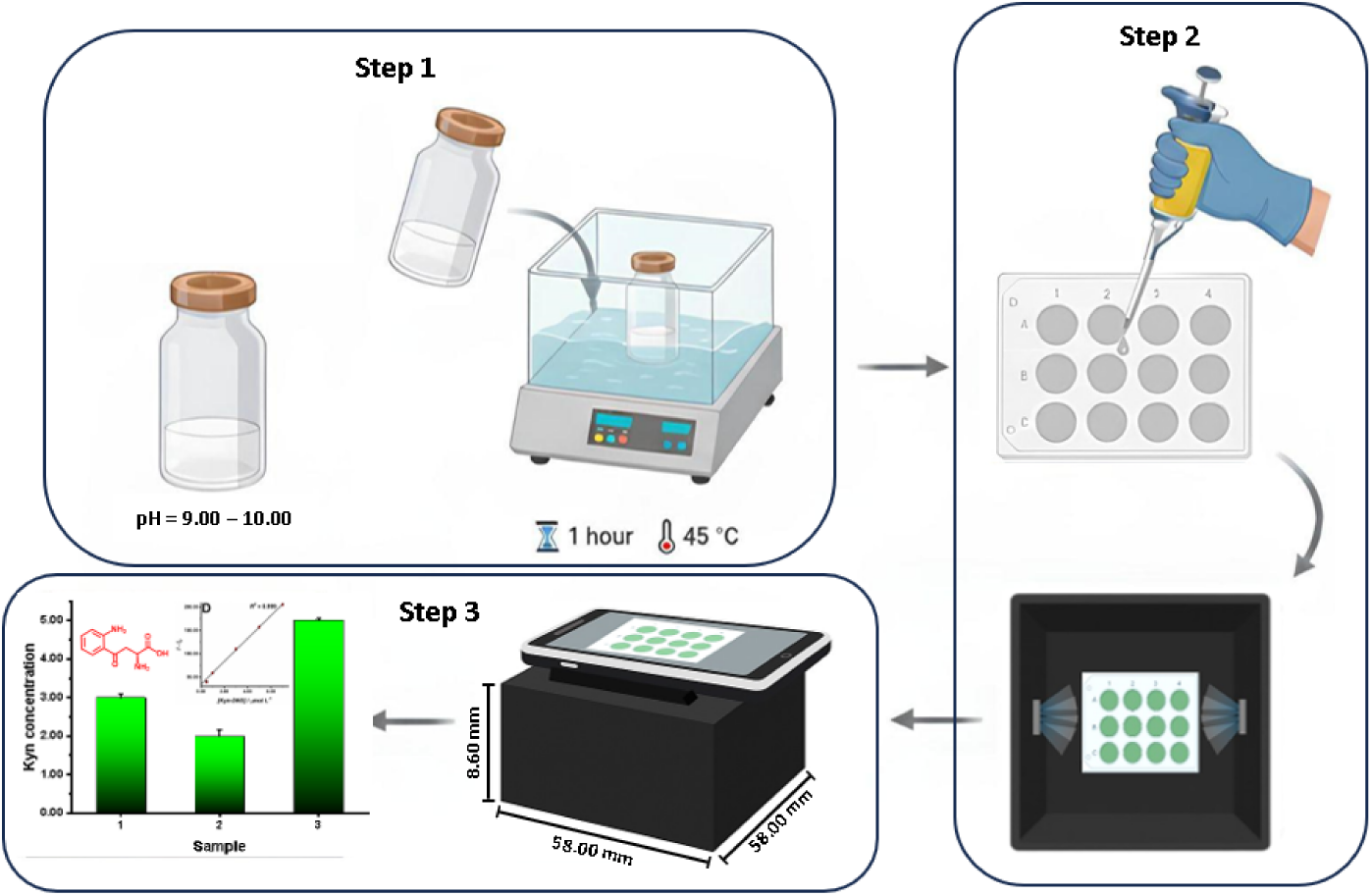
Procedure for quantifying Kyn using the Kyn-DNS/3Dchamber/FDIB method proposed. Step 1: Derivatization reaction in Kyn solutions and samples with DNS-Cl to form the Kyn-DNS fluorescent marker. Step 2: Excitation of the Kyn-DNS adduct in the chamber using UV-LEDs; and Step 3: Acquisition of fluorescence images using the chamber with the smartphone coupled to it, followed by data processing for quantitative purposes.

### 2.6 Analytical Validation Parameters

Analytical curves were constructed over the concentration range of 0.50 to 7.00 μmol L^–1^ to quantify Kyn in saliva, with data in triplicate. The figure merits were calculated following well-established analytical chemistry guidelines and procedures previously adopted by our group.^26,29,33^ The limit of detection (LOD) and limit of quantification (LOQ) were calculated based on the standard deviation of the blank signal (σblank) and analytical sensitivity (S), according to eq 1 and 2:

2 LOD = (3 ×σblank) / S

and

3 LOQ = 3.3 × LOD

The blank standard deviation (σblank) was obtained from n = 3 independent measurements of the blank solution, acquired under the same experimental and image acquisition conditions as the analytical measurements. The σblank value was calculated as the standard deviation of the mean RGB intensity values extracted from the selected region of interest (ROI). The analytical sensitivity (S) was obtained from the slope of the analytical calibration curve, constructed using the graph of the corrected RGB signal as a function of analyte concentration,^26,29,40^

The repeatability of the analytical method was evaluated at three concentration levels (0.50, 3.00, and 7.00 μmol L^–1^) by performing repeated analyses over three consecutive days under consistent conditions with regard to protocol, equipment, environment, and analyst. All tests were performed in triplicate (*n* = 3). Variability was expressed as the coefficient of variation (CV), calculated according to,

4 $$CV\%=\frac{SD}{\mathrm{x̅}}\times 100\%$$

where *SD* is the standard deviation and *x̅* is the mean of the values obtained.

To evaluate the matrix effect and method accuracy, the recovery test was performed using fixed concentrations of 0.50 and 1.00 μmol·L^–1^ Kyn. Recovery was calculated using the eq 3:

5 $$\mathrm{Recovery}\left(\%\right)=\frac{\left(C_{1}-C_{2}\right)}{C_{2}}\times 100\%$$

where *C*_1_ is the concentration found, and *C*_2_ is the added concentration.^26^ To determine the accuracy of the method, samples fortified at the same concentrations as in the previous test were quantified using a spectrofluorometry method. An *F*-test was conducted to assess variance homogeneity, while the Student’s *t*-test was used to determine whether the mean values obtained from both methods were statistically equivalent for a 95% confidence level. The interference test was performed with species normally found in saliva samples (Kyna, Quina, Pic, Ura, and citrate).^1–6^ Kyn was derivatized at a fixed concentration of 3.00 μmol L^–1^ in the presence of possible interferents in three ratios (1:1, 1:10, and 1:100) (Kyn: interferents), and the relative error was calculated.

## 3 Results and Discussion

### 3.1 Fluorescence Derivatization Reaction of Kynurenine

Due to the presence of the amino aromatic group, Kyn was derivatized with DNS-Cl into a fluorescent product, Kyn-DNS. The derivatization reaction of Kyn with DNS-Cl occurred under optimized conditions, in an alkaline medium (pH = 9.00–10.00), and in a water bath at 45.0 °C for 1 h (Section 2.3). The fluorescence of the Kyn-DNS product was then investigated, where the emission fluorescence spectra of Kyn and the Kyn-DNS product were recorded after excitation at 365 nm (Figure 4). The fluorescence emission spectra of the Kyn-DNS solution clearly demonstrated that this product exhibited a high fluorescence compared to the control, DNS-Cl prepared in acetonitrile, which has no fluorescence emission bands in the absence of water, with a significant increase in the emission band at 508.30 nm, (Figure 4A). A similar behavior was observed when the derivatization reaction occurred in the artificial saliva sample (Section 2.3) and the Kyn-DNS product demonstrated, as expected, a significant increase in fluorescence compared to the control (Figure 4B).

**Figure 4:**
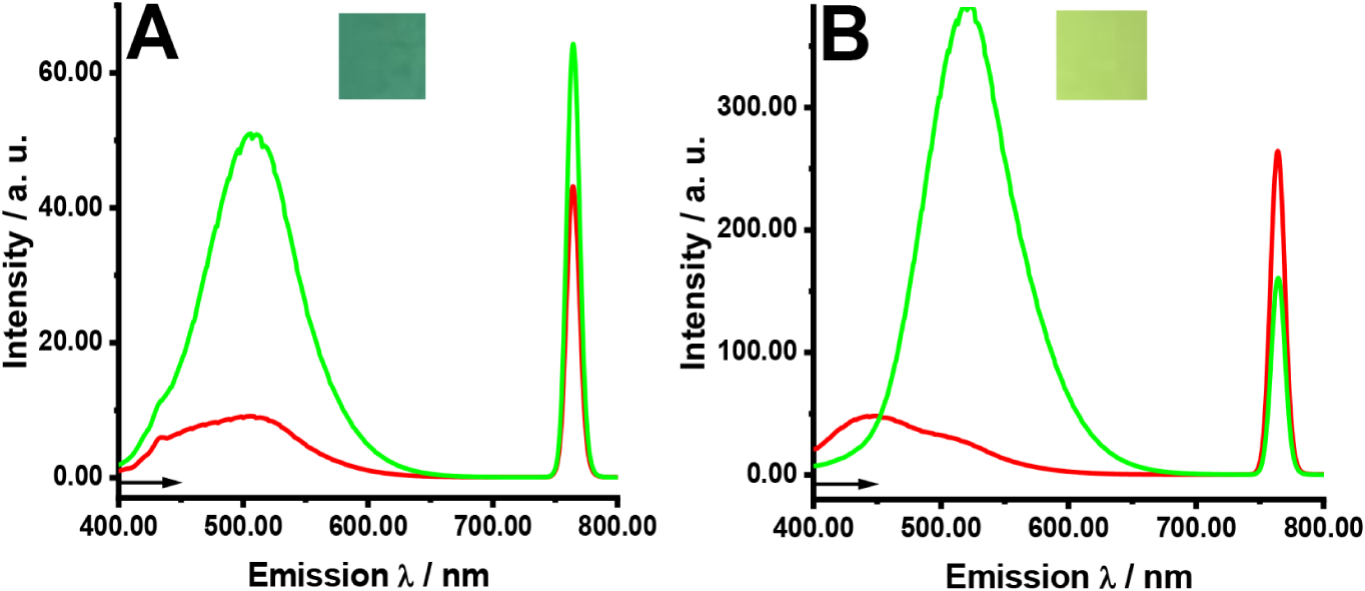
Fluorescence emission spectra after excitation at 380.00 nm of (A) 5.00 μmol L^–1^ Kyn-DNS product in acetonitrile and (B) in a saliva sample from a derivatization reaction control (DNS-Cl) (Red line) and Kyn-DNS product (Green line). Inset figures: digital image captured by FDIB after derivatization.

To verify the kinetics and stability of the derivatization reaction of Kyn with DNS-Cl, the fluorescence emitted by the Kyn-DNS product was monitored for 1 h at different time intervals using the FDIB method proposed (Figure S1). The results showed that the fluorescence emitted by the Kyn-DNS product remains stable, with small variations over time (RSD of <5%). For this reason, a minimum time of 30 s was chosen for the derivatization reaction in the proposed FDIB method to obtain a sensitive, precise, and fast approach. Moreover, digital images of the fluorescence of Kyn-DNS products were captured with a smartphone in the proposed reaction chamber to corroborate the fluorescence spectra presented in Figure 4. Thus, similar to the fluorescence spectra that showed a highly intense signal at 500 nm (green), the FDIB presented an intense fluorescence in the green channel (G) from the RGB system (Figures 5 and 6). The second band from the spectrofluorescence spectra, between 750 nm and 800 nm (near-infrared band) is close to the red (R) final domain from the RGB system (550–750 nm),^62,63^ and thus, it is expected a lower sensitivity for the R channel compared to the green channel in the FDIB method,^40^ as presented in Figure 6 and Table 1. Following the protocol, no subsequent procedures were performed to remove excess DNS-Cl, reaction byproducts, or to perform purification, extraction, or filtration procedures, since this approach makes the method faster and simpler than those previously reported in the literature, where these additional steps were included to eliminate excess DNS-Cl, which fluoresces in aqueous media and can lead to interference.^26,45,47–50^

**Figure 5:**
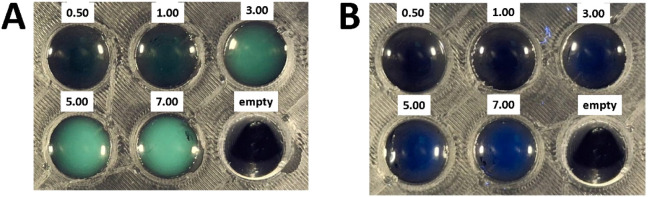
Digital images of fluorescence obtained by smartphone coupled to the 3D chamber for solutions of different concentrations of (A) Kyn-DNS and (B) control (DNS-Cl).

**Figure 6:**
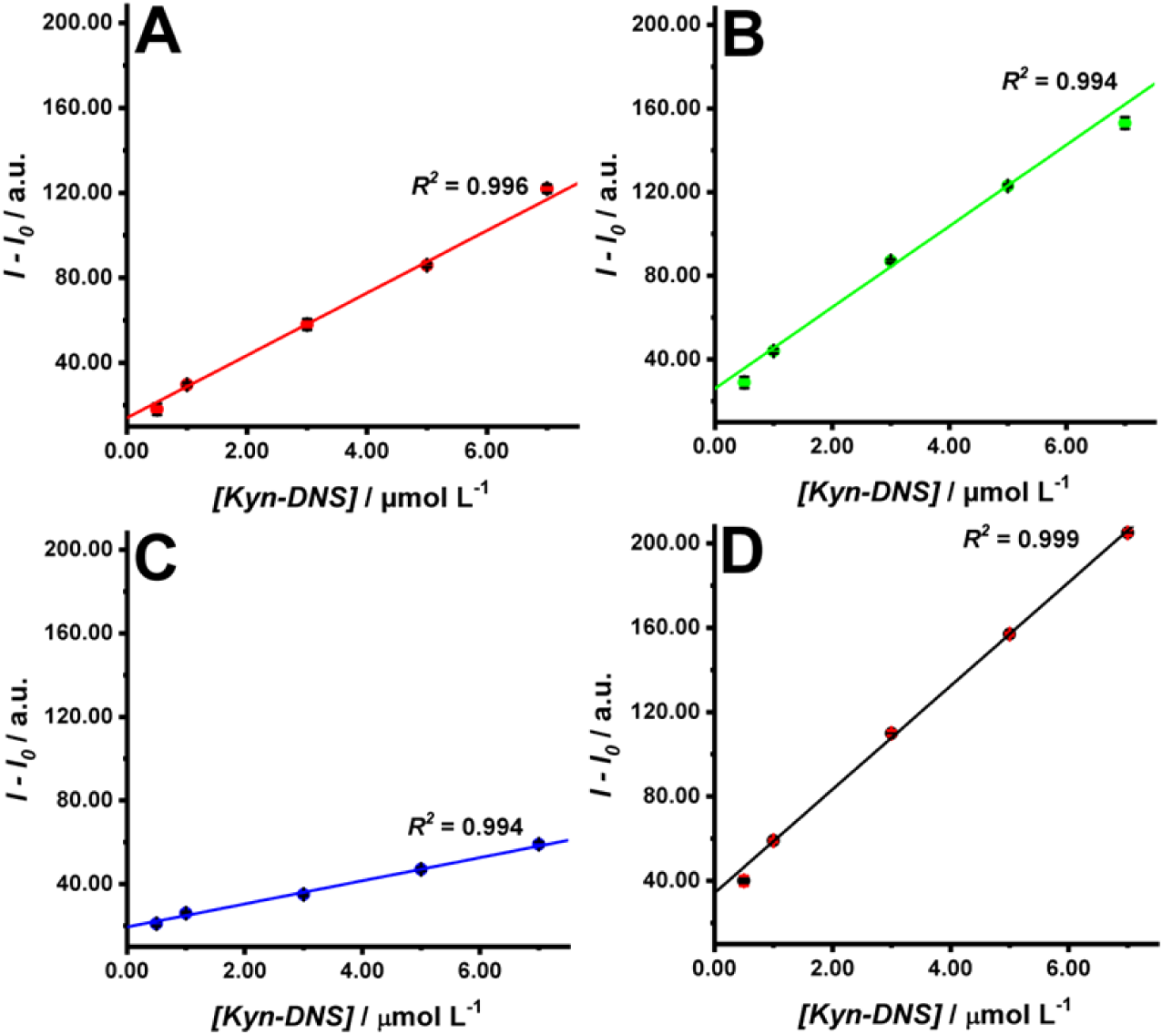
Plots of the analytical curves by the Kyn-DNS/3Dchamber/FDIB method proposed here for the (A) red, (B) green, (C) blue, and (D) vector channels vs Kyn-DNS concentration. Error bars represent the standard deviation for three independent measurements.

**Table 1:** Parameters Obtained for the Analytical Curves Using the Kyn-DNS/3Dchamber/FDIB Method

Parameters	Red	Green	Blue	Vector
**Linear range (μmol L** ^**–1**^)	0.50–7.00	0.50–7.00	0.5–7.00	0.5–7.00
**LOD (μmol L** ^**–1**^)	0.13	0.15	0.98	0.05
**LOQ (μmol L** ^**–1**^)	0.41	0.49	3.24	0.15
**Sensitivity (a.u. μmol L** ^**–1**^)	14.69 (± 1.99)	19.41 (± 0.85)	5.53 (± 0.25)	24.54 (± 0.39)
**Linear coefficient (a.u)**	14.14 (± 0.52)	26.09 (± 3.28)	19.43 (± 0.89)	34.24 (± 1.61)
***R*** ^**2**^	0.996	0.994	0.994	0.999

The 3D black chamber allowed the acquisition of low levels of spurious signals. Indeed, the low background signal is due to the low intensity of the signal emitted by the UV-LED in the visible spectrum (Figure S2). Indeed, the spurious signals were 5.15 (±0.16), 5.62 (±0.32), and 25.01 (±0.19) for the R, G, and B channels, respectively (Figure S2). These values compared to 255 (full scale) for each channel were less than 10%. However, for better accuracy, all spurious data were subtracted from the analytical signal, making them insignificant, and an excellent precision ≤5.75% was obtained. This assessment is necessary since high spurious signals can result in interference with the analytical signal. To determine the best software for RGB data acquisition, the ImageJ software for Windows and the Color Grab app for smartphones were evaluated. Figure S3 was analyzed using both ImageJ and the Color Grab app, obtaining similar precision with a relative standard deviation (RSD) of less than 2.26%. *t*-tests and F-tests were applied at a 95% confidence level with *n* = 3. No significant difference was found between the two software programs at a 95% confidence level (Table S1). Thus, we used the Color Grab app for smartphones due to its high portability.

### 3.2 Fluorescence Digital Image-Based Method

After verifying the efficiency of the Kyn derivatization reaction, the FDIB method was used to obtain analytical curves for the determination of Kyn in artificial saliva samples. As mentioned above, digital images from a smartphone, using a 3D chamber with LEDs at 365 nm, indicated that the Kyn-DNS product exhibited fluorescence emission close to green, while the control, DNS-Cl, emitted light close to blue (Figure 5). The blue fluorescence of the DNS-Cl in aqueous solution is a typical result reported in the literature, but here, this fluorescence was not a problem because other channels from the RGB system supply a satisfactory response, such as the green and red channels.^45,47–49^ Thus, analytical curves were obtained with different concentrations of Kyn-DNS vs the RGB signals obtained in the digital images (Figure 5A). For all concentrations, the control signal was subtracted (*I* – *I*_0_) (Figure 6). The difference in the intensity of the blue color due to the DNS-Cl is because the increase in its concentration is proportional to the increase in Kyn to maintain the 1:1 molar ratio, avoiding the use of excess reagent as required for green chemistry purposes. The analytical curves recorded indicated excellent linearity in the relationship between the concentration of the Kyn-DNS derivative product and its fluorescence in each RGB color channel (Figure 6). The channels showed distinct linear responses as a function of Kyn-DNS concentration, with differences in sensitivity clearly noted in the slopes of the respective calibration equations (Figure 6).

In the red channel (Figure 6A), the equation (*I* – *I*_0_) = 14.69 (±1.99) *[Kyn-DNS]* (μmol L^–1^) + 14.14 (±0.52) presented a good sensitivity and a high determination coefficient (*R* ^2^ = 0.996). The green channel (Figure 6B) exhibited a higher slope compared to other channels (*I* - *I*_0_) = 19.41 (±0.85) *[Kyn-DNS]* (μmol L^–1^) + 26.09 (±3.28), and *R* ^2^ = 0.994, indicating greater sensitivity and a good model fit, as expected due to the yellow-green emission.^45,47–49^ Although the blue channel showed a good *R* ^2^ value of 0.994, it had a lower slope (*I* – *I*_0_) = 5.53 (±0.25) *[Kyn-DNS]* (μmol L^–1^) + 19.43 (±0.89) and therefore lower sensitivity in comparison with the green channel (Figure 6C), mainly due to the emission of DNS in an aqueous medium. This behavior is related to the fluorescence of derivatized Kyn-DNS, which is shifted toward green with a higher signal intensity (Table 1). In the case of the response obtained from processing the data using the RGB vector (Figure 6D), which combines the three channels, the resulting calibration curve was described by the equation (*I* – *I*_0_) = 24.55 (±0.39) *[Kyn-DNS]* (μmol L^–1^) + 34.24 (±1.61), exhibiting the highest sensitivity and *R* ^2^ among all curves, *R* ^2^ = 0.999. This behavior demonstrates that using the RGB vector enhances the analytical response by combining the spectral information from the individual channels, thereby increasing the robustness of the method for Kyn quantification (Table 1 and Table S2). Thus, the vector was chosen for Kyn quantification. These results corroborate with the fluorescence emission spectra shown in Figure 4.

### 3.3 Analytical Validation Parameters

In this study, the LOD and LOQ of the proposed method were estimated using the standard deviation of the blank signal and the analytical sensitivity obtained from the calibration curve. The standard deviation of the blank (σblank), calculated from n = 3, was 0.381 (a.u.). The analytical sensitivity, expressed as the slope of the calibration curve constructed from the corrected RGB vector signal, was 24.54 (a.u. μmol L^–1^). Based on these parameters, the LOD and LOQ of the proposed method were determined to be 0.05 μmol L^–1^ and 0.15 μmol L^–1^, respectively. These values are among some of the lowest reported in the literature for alternative methods to detect Kyn (Table 2). This performance highlights the suitability of the proposed Kyn-DNS/3Dchamber/FDIB method for applications requiring Kyn trace detection, since the LOD is lower than the Kyn levels found in healthy people groups, ranging from 0.50 to 0.70 μmol L^–1^ and for clinical patients with different pathologies, ranging from 2.31 to 3.45 μmol L^–1^.^7,8^ The low LOD and LOQ values also reinforce its potential as a reliable alternative to conventional techniques such as spectrofluorometry, HPLC-FD, LC-UV/FD, LC-MS, and the FDIB presented compatible sensitivity to electrochemical methods, Table 2.

**Table 2:** Comparison of Some Analytical Methods for Determination of Kyn in Biological Fluids with the Kyn-DNS/3Dchamber/FDIB Method Proposed^a^

Method	Linear range (μmol L^–1^)	LOD (μmol L^–1^)	ref.
Chromatographic methods			
HPLC–FD	0.18–2.90	0.03	^12^
LC–UV/FD	0.24–2.88	0.06	^14^
LC–MS	0.41–7.00	0.001	^15^
Electrochemical methods			
DPV–Bif-BDDE	0.10–20.00	0.03	^18^
DPP–HMDE	0.20–30.00	0.03	^20^
DPV–aGCE	2.00 – 8.62	0.43	^19^
Optical methods			
Chemosensor–FD	1.00–20.00	0.70	^13^
FDIB–Smartphone	0.50 – 7.00	0.05	This work

a Abbreviations used: high-performance liquid chromatography (HPLC), fluorescence detection (FD), bismuth film-modified boron-doped diamond electrode (Bif-BDDE), differential pulse polarography (DPP), differential pulse voltammetry (DPV), hanging mercury drop electrode (HMDE), liquid chromatography (LC), electrochemically pretreated glassy carbon electrode (aGCE), mass spectrometry (MS), and fluorescence digital image-based (FDIB).

The repeatability test of the Kyn-DNS/3Dchamber/FDIB proposed method showed that the coefficient of variation remained below 2.68% at all evaluated Kyn concentration levels: 0.50 μmol L^–1^ (Figure 7), 3.00 μmol L^–1^ (Figure S4A), and 7.00 μmol L^–1^ (Figure S4B). The relative standard deviations (RSD) were all below 6.93% (Table S3), confirming that the methodology provides the required precision.^26,29,40^ These values demonstrate low variability among replicates, both intra- and interday, confirming the robustness of the Kyn-DNS/3Dchamber/FDIB method proposed for Kyn determination. The accuracy of the proposed method using Kyn-DNS/3Dchamber/FDIB was also evaluated by recovery testing and comparing it with data obtained from conventional analysis using spectrofluorometry (Section 2.4). The recovery test results were satisfactory and ranged from 92.1% to 106%, which is better than the reference method using spectrofluorometry (Table 3). Both methods yielded satisfactory accuracy results, considering the recovery range from 80.0 to 120.0%.^26,29,40^ This result is very interesting because the synthetic saliva is a complex sample containing beef extract, yeast extract, protease peptone, hog gastric mucin, NaCl, KCl, CaCl_2_, and urea. Thus, these samples were used to evaluate the robustness of the method.

**Figure 7:**
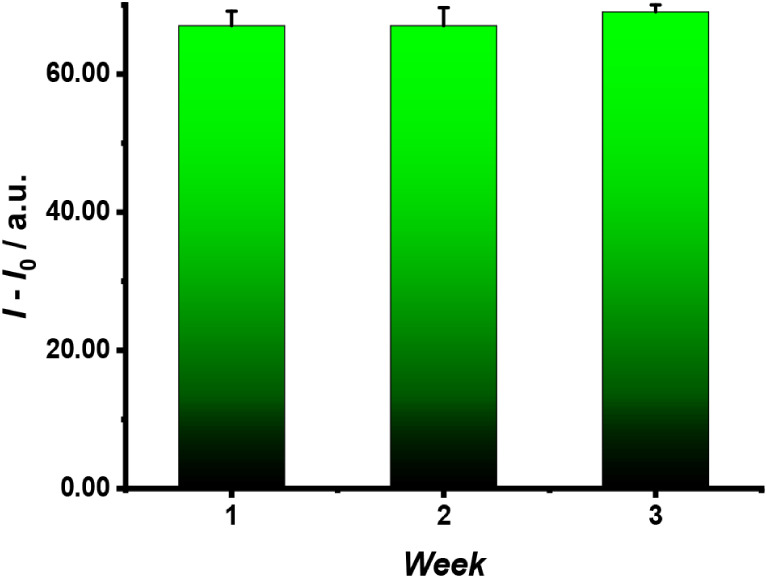
Repeatability tests, both intra- and interday, of the proposed Kyn-DNS/3Dchamber/FDIB method for the RGB vector of 0.50 μmol L^–1^ Kyn-DNS.

**Table 3:** Recovery Assay of the Kyn-DNS/3Dchamber/FDIB Proposed Method Compared with the Spectrofluorometry Technique Used as Reference

Sample	Added/μmol L^–1^	FDIB: Found ± SD (Recovery %)	Reference: Found ± SD (Recovery %)	*t* test	F-test
S1	0.50	0.50 ± 0.03 (100%)	0.44 ± 0.09 (88.6%)	0.67	3.00
	1.00	1.00 ± 0.03 (100%)	0.90 ± 0.04 (90.0%)	1.27	1.33
S2	0.50	0.46 ± 0.03 (92.1%)	0.40 ± 0.02 (79.0%)	1.29	1.50
	1.00	1.10 ± 0.04 (106%)	0.99 ± 0.07 (98.7%)	1.02	1.75
S3	0.50	0.46 ± 0.05 (92.6%)	0.41 ± 0.09 (81.5%)	0.61	1.80
	1.00	0.97 ± 0.04 (97.4%)	0.84 ± 0.04 (84.1%)	1.42	1.00

A statistical comparison between the Kyn-DNS/3Dchamber/FDIB proposed method and the spectrofluorometry technique did not reveal significant differences between the mean values obtained, as the *t*_calculated_ values were lower than the *t*_critical_ value (4.303), indicating that the two methods are statistically equivalent for 95% confidence level.^26,29,40^ In addition, the *F*-test confirmed the homogeneity of variances, with *F*_calculated_ values lower than *F*_critical_ (19.00), Table 3.^26,29,40^ Similar results were obtained using the HPLC-UV method. The chromatograms from standard solutions and samples, along with the calibration curve, are presented in Figure S5 as well as the recovery results and statistical data (Table S4). These results demonstrate that the FDIB method is a precise and accurate method for determining Kyn in saliva samples.

To evaluate the method’s selectivity against potential interferences present in saliva, tests were carried out together with other organic compounds. The compounds evaluated were Kyna, Quina, Pic, Ura, and citrate. The first three compounds are additional metabolites of the KynP, which have been investigated for their bioactive properties and their analytical potential as biomarkers of neurodegenerative and inflammatory diseases.^1–6^ Another species evaluated was Ura, a metabolite of purine degradation, studied as a biomarker for metabolic diseases, oxidative stress, and other associated diseases.^62^ Citrate, an anion that is a byproduct of several metabolic pathways in the body and is produced in saliva by the salivary glands, was also studied.^63^ In interference tests, the relative error (RSD) ranged from 0.51% to 9.75% (Table 4) values within the ±15.00%, demonstrating a good selectivity.^26,29,40^ At a ratio of 1:100 Ura, the RSD of 5.32% suggests that during the derivation reaction, the −NH groups of the Ura compete with the reactive sites of Kyn, but no significant interference occurs (Table 4). Fluorescence spectra were obtained for the 1:1 ratio of Kyn and possible interferents (Figure S6). The results showed, as verified for the values obtained with the FDIB method, that there was no significant interference, since, according to the spectra, there was no change for the Kyn-DNS emission band. A little bit change in the fluorescence was due to the effects of dilution due to the addition of the solution into the control solution containing Kyn-DNS.

**Table 4:** Interference Study of Metabolites on the Determination of Kyn in Artificial Saliva

Interferent	Kyn:interferent ratio	Relative error
Ura	1:1	0.49%
	1:10	–1.92%
	1:100	5.32%
Kyna	1:1	0.83%
	1:10	3.11%
	1:100	4.07%
Quina	1:1	0.48%
	1:10	4.95%
	1:100	9.15%
Pic	1:1	–0.51%
	1:10	3.79%
	1:100	–5.10%
Citrate	1:1	–3.00%
	1:10	–3.54%
	1:100	–9.75%

The synthetic saliva is a complex mixture of compounds containing beef extract, protease peptone enzyme, yeast extract, hog gastric mucin type III presented as a branch of protein, amino acids, and potential interference compounds, and no significant interference was obtained according to recovery tests and interferent results.^52,57–61^ Probably, the time, temperature, and pH optimized in the represented methods are not preferred for these compounds compared to Kyn.^26,52,61^ Indeed, it is verified that the derivatization reaction using DNS-Cl and its selectivity are influenced by the following reaction conditions: reaction time, temperature, pH of the medium, and DNS-Cl concentration.^26,52^ Thus, different target analytes, since amino acids, proteins, biogenic amines, and Kyn with different p*K*_a_ values can be selected allowing an increase in selectivity.^26,52,61^ The results for the derivatization reaction are consistent with the literature, since Kyn presents three p*K*_a_ values in its structure: pKa1 = 1.19, pKa2 = 2.49, and pKa3 = 9.33,^61^ and thus, the amino groups of Kyn are deprotonated at pH 10, which was chosen to enable the reaction with DNS-Cl, as demonstrated in previous works.^26,52,61^ This selectivity was also checked and validated by chromatography results, Figure S5 and Table S4.

The validation parameters demonstrated that the Kyn-DNS/3Dchamber/FDIB method has high accuracy, precision, and repeatability, combined with low detection limits (LOD) and quantification limits (LOQ). Good selectivity was also observed, evidenced by the method’s ability to differentiate the analyte from possible interferents present in the matrix. These results confirm the robustness of the method, which, in addition to satisfactory analytical performance, is in line with the principles of Green Chemistry, as it uses reduced amounts of reagents, generates low volumes of waste, and requires only 150.00 μL to perform the analyses. While the use of synthetic saliva allowed for a highly controlled environment for analytical methods validation, the artificial backgrounds may not fully capture the biochemical complexity of clinical samples.^52,57–61^ Factors such as varied protein concentrations and individual microbiota present in biological saliva could influence the FDIB method’s accuracy.^52,57–61^ On the other hand, the current results provide a robust proof-of-concept; further biological analysis can be performed for future applications.^64,65^

### 3.4 Green Metrics

To verify whether the Kyn-DNS/3Dchamber/FDIB proposed method was aligned with the green chemistry proposal and considered an environmentally friendly detection tool, it was subjected to a careful analysis using three widely used metric tools. Generally, green metrics use common parameters such as the type of reagent used, energy consumption, reagent toxicity, sample pretreatment steps, use of derivatization, and so on. The first tool used was the GREENness analytical calculator,^66,67^ which is based on the application of 12 principles, as described by F. Pena-Pereira, W. Wojnowski, and M. Tobiszewski and listed in Table S5.^66^ According to this metric system, the central values (inside the circle) range from 0.00 to 1.00, where 0.00 represents a method with low green characteristics and 1.00 represents a highly green method, thus being environmentally friendly. Figure 8A shows that the FDIB method proposed in this work demonstrated remarkable performance, exhibiting green characteristics (value = 0.79), when compared to the spectrofluorometry method (value = 0.56), for quantifying Kyn, Figure 8B. The penalty observed in principles 6, 10, and 11 refers to the use of derivatization using DNS-Cl.

**Figure 8:**
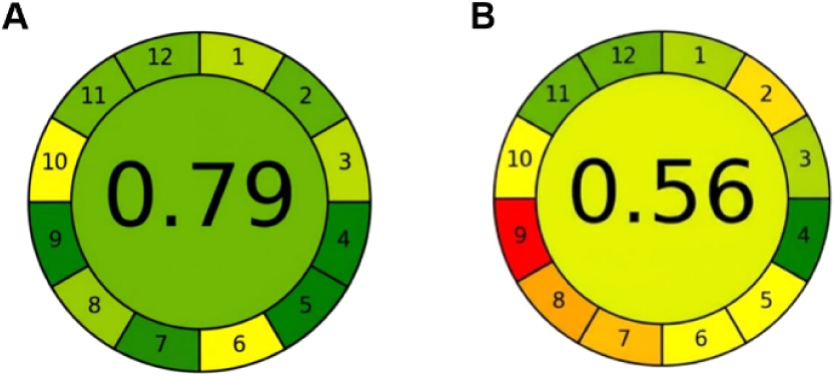
GREENness calculator with score of the (A) FDIB proposed method and the (B) method using spectrofluorometry.

The second metric tool employed was the GAPI (Green Analytical Procedure Index), proposed by Płotka-Wasylka is represented by a pictogram composed of five colored pentagons in shades of red, yellow, and green, representing high environmental impact, medium environmental impact, and low environmental impact, respectively.^66,68^ The five pentagons represent different stages of the analytical metric, namely: sample preparation, chemical reagents used, sampling, type of methods, and instrumentation. The parameters used by the GAPI method are described in detail in Table S6, and Figure 9 shows that the FDIB method demonstrated excellent performance, as evidenced by the predominant green color in almost all requirements, with the exception of the pentagon corresponding to the reagents used in the analysis.

**Figure 9:**
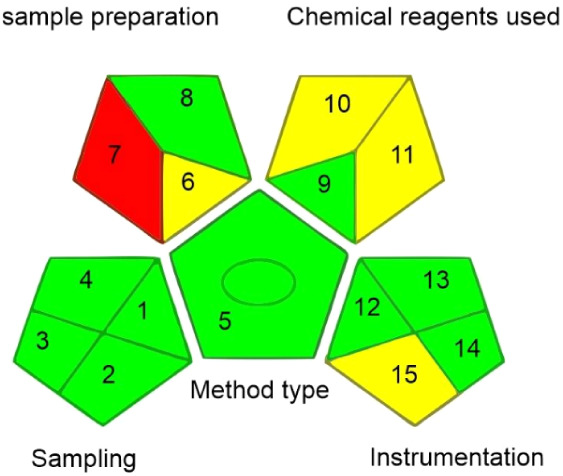
GAPI metric pictograms for the FDIB proposed method.

The third tool used to analyze the proposed method was the BAGI (Blue Applicability Degree Index), a metric proposed by Manousi et al., which can be calculated on its official website: https://bagi-index.anvil.app/.^69^ The BAGI uses a color scale and a star-shaped pictogram to assess the method’s feasibility and analytical performance. Being a relatively simple metric, the BAGI score ranges from 25.0 to 100.0, with the closer to 100.0, the more practical the method. The BAGI metric uses ten criteria, which are detailed in Table S7. As shown in Figure 10, the FDIB method achieved an excellent score, value = 77.5, demonstrating its practicality. It was penalized only for the reagents used and the presence of the derivatization step. The penalties were not due to the FDIB method itself but rather to the use of DNS-Cl and the derivatization step required for the analysis of Kyn. Thus, the proposed FDIB method demonstrated excellent performance when analyzed using the GREENness, GAPI, and BAGI metrics, demonstrating its environmental friendliness, alignment with green chemistry policies, and practical and easy-to-use approach. It is an attractive alternative to conventional analytical methods that require complex, high-power, energy-intensive instrumentation, toxic solvents, and sample pretreatment.

**Figure 10:**
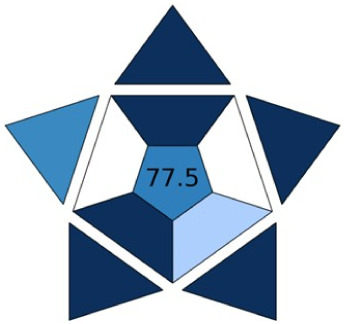
BAGI metric pictograms for the FDIB proposed method.

## 4 Conclusions

For the first time in the literature, a method for the detection and quantification of kynurenine (Kyn) using the fluorescence digital image-based method Kyn-DNS/3Dchamber/FDIB was developed. The Kyn-DNS/3Dchamber/FDIB method yielded highly satisfactory results, enabling the determination of concentrations below 7.00 μmol L^–1^ in artificial saliva samples, with good precision, accuracy and recovery values ranging from 92.1% to 106%. Indeed, the method presented good agreement at a 95% confidence level when compared to spectrofluorometry and HPLC-UV methods. Furthermore, the method proved to be practical, simple, and rapid, requiring only a few analytical steps and a small amount of reagents, 150.00 μL per analysis in approximately 30 s, much less than reference methods. Green chemistry metrics show that the FDIB method is aligned with green analytical chemistry. The FDIB method, therefore, presents potential applications for the determination of Kyn in noninvasive biological fluids such as saliva, given its accessibility, feasibility, and cost-effectiveness, as it combines analytical innovation with environmental, economic, and public health considerations. Furthermore, the method can be used for fast point-of-care analysis; samples can be easily collected and analyzed on site, since the necessary instrumentation is simple, portable, low-cost, and widely accessible.

## Supplementary Material



## Notes

Results of repeatability tests intra- and interday of the proposed Kyn-DNS/chamber3D/FDIB method for the RGB vector for three concentrations of Kyn; HPLC-UV results; principles employed by the following green metrics: AGREE, GAPI, and BAGI (PDF)
