# Supplementary material for: Quantification of the Kynurenine Biomarker in Saliva Using Smatphone-Based Fluorescence Digital Imaging
Source: ACS Omega. 2026 Jun 15;11(25):36627–38. doi: 10.1021/acsomega.5c11977 (PMC13325347; doi:10.1021/acsomega.5c11977)
Supplement: Supplementary file 1 [file ao5c11977_si_001.pdf]

Supplementary Information

Quantification of the Kynurenine Biomarker in Saliva Using Fluorescence Digital Image by Smartphone

José Gouveia da Silva Neto[a,b], Luís Vinícius Gonçalves de Melo[a], José Ailton Mota Nascimento[b], Helayne S. de Sousa[a], João Paulo de Almeida[a], Severino Carlos Oliveira[b]* and Vagner Bezerra dos Santos[a]*

[a]Department of Fundamental Chemistry, Federal University of Pernambuco, Recife, 50740-560, PE, Brazil (Av. Jornalista Anibal Fernandes, S/N, Cidade Universitária)
[b]Department of Chemistry, Federal Rural University of Pernambuco, Recife, 52171-900, PE, Brazil (Dom Manuel de Medeiros Street, S/N, Dois Irmãos)

Corresponding author:
vagner.bsantos@ufpe.br / Recife, 50740-560, PE, Brazil (Av. Jornalista Anibal Fernandes, S/N, Cidade Universitária)
severino.oliveira@ufrpe.br / Recife, 52171-900, PE, Brazil (Dom Manuel de Medeiros Street, S/N, Dois Irmãos)

Table of Contents

Figure S1. Fluorescence signal for the vector channel of the 60.00 µmol L^{-1} Kyn-DNS product, Measurements at different times (min) after the derivatization reaction. S1
Figure S2. RGB data values for the spurious signal from the 3D black chamber. S1
Figure S3. Digital images of fluorescence obtained by smartphone coupled to the 3D chamber for a solution of 60.00 µmol L^{-1} Kyn-DNS. S1
Figure S4. Repeatability tests intra and inter-day of the proposed Kyn-DNS/chamber3D/FDIB method for the RGB vector of (A) 3.00 and (B) 7.00 µmol L^{-1} Kyn.. S2
Figure S5. Chromatogram from HPLC-UV for detection of Kyn at 360 nm (A). The calibration curve using the area is inserted as Figure (B). The linear range was from 0.5 to 7.0 µmol L-1 Kyn. S3
Figure S6. Fluorescence emission spectra of 3.00 µmol L^{-1} Kyn-DNS product with possible interferes in a 1:1 ratio, from the derivatization reaction. S3
Table S1. RGB data acquisition by Imagej software compared with the Color Grab app. S4
Table S2. RGB data, I-I_0 (a.u.) for the analytical curves using the Kyn-DNS/3Dchamber/FDIB method. S4
Table S3. Relative error for the repeatability test inter-day for Kyn. S5
Table S4. Recovery assay of the Kyn-DNS/3Dchamber/FDIB proposed method compared with the HPLC-UV technique. S6
Table S5. Principles used by the metric system of the GREENess analytical calculator. S6
Table S6. Principles employed by the GAPI metric system.. S7
Table S7. Principles employed by the BADI metric system. S8

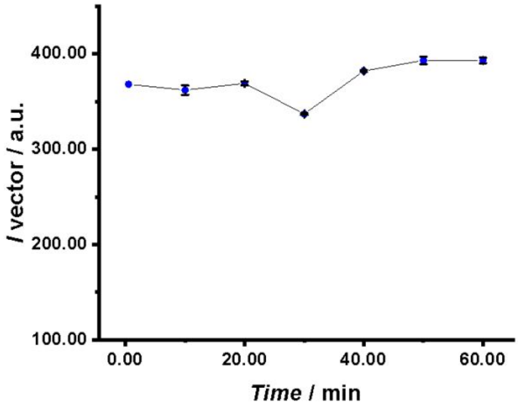

Figure S1. Fluorescence signal for the vector channel of the 60.00 µmol L^{-1} Kyn-DNS product, measurements at different times (min) after the derivatization reaction.

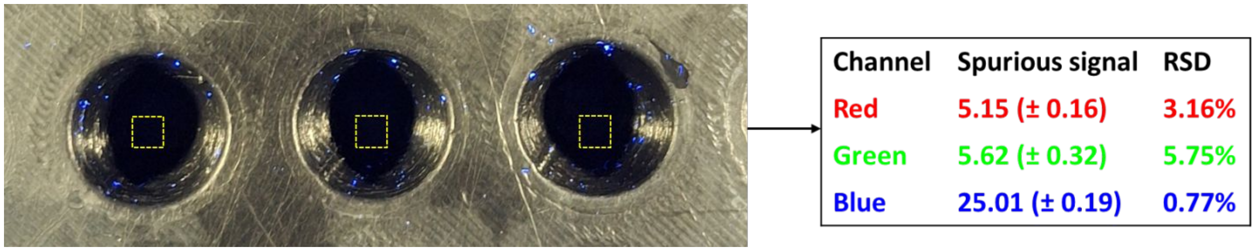

Channel	Spurious signal	RSD
Red	5.15 (± 0.16)	3.16%
Green	5.62 (± 0.32)	5.75%
Blue	25.01 (± 0.19)	0.77%

Figure S2. RGB data values for the spurious signal from the 3D black chamber.

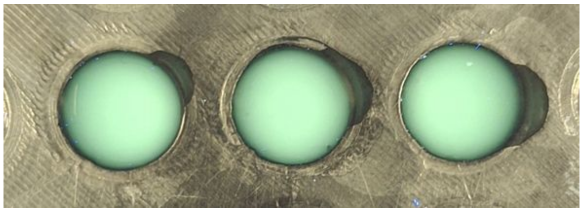

Figure S3. Digital images of fluorescence obtained by smartphone coupled to the 3D chamber for a solution of 60.00 µmol L^{-1} Kyn-DNS for the formation of the Kyn-DNS fluorescent marker.

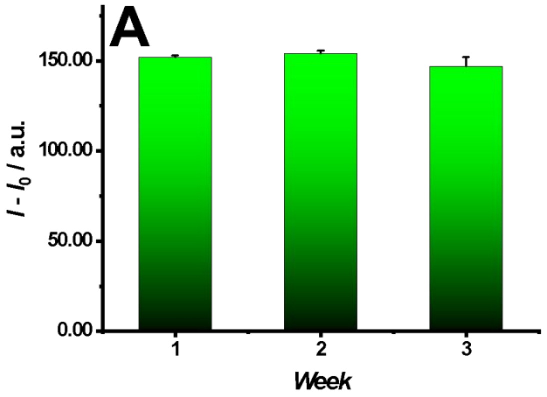

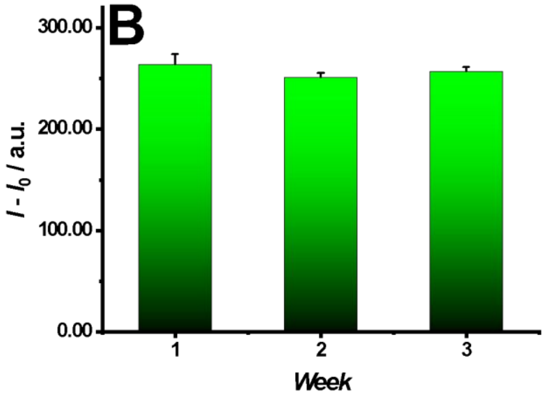

Figure S4. Repeatability tests intra and inter-day of the proposed Kyn-DNS/chamber3D/FDIB method for the RGB vector of **(A)** 3.00 and **(B)** 7.00 μmol L^{-1} Kyn-DNS.

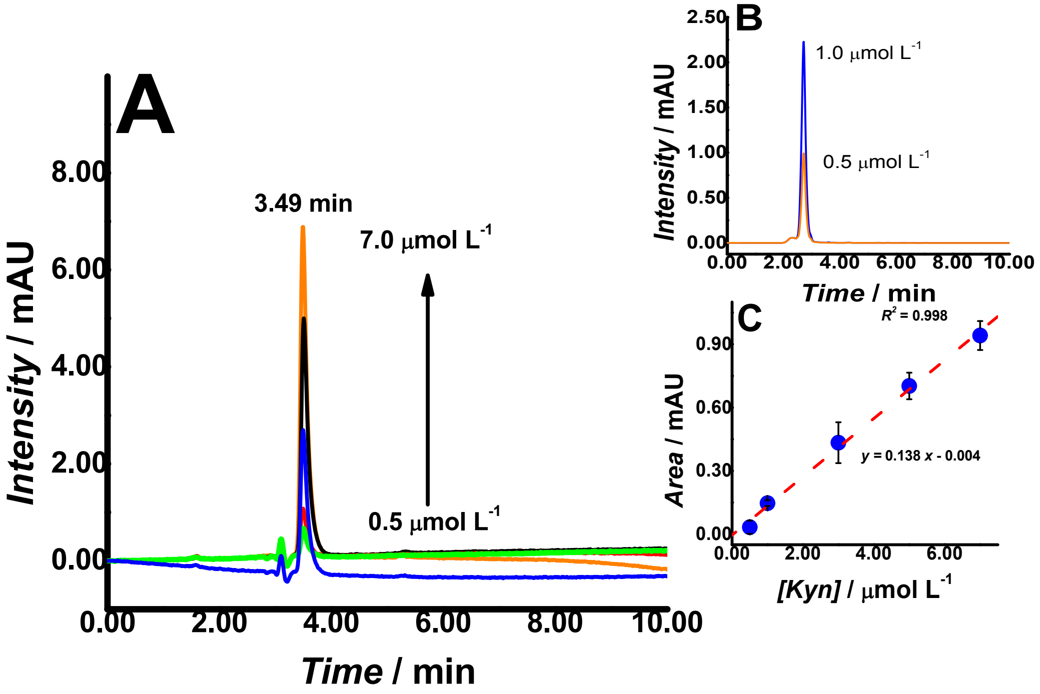

Figure S5. Chromatograms from HPLC-UV for detection of Kyn at 360 nm (A). Chromatograms from Synthetic Saliva (S1) spiked with 0.5 and 1.0 µmol L^{-1} Kyn (B). The calibration curve using the area used to quantify Kyn (C). The linear range of the HPLC-UV method was from 0.5 to 7.0 µmol L^{-1} Kyn.

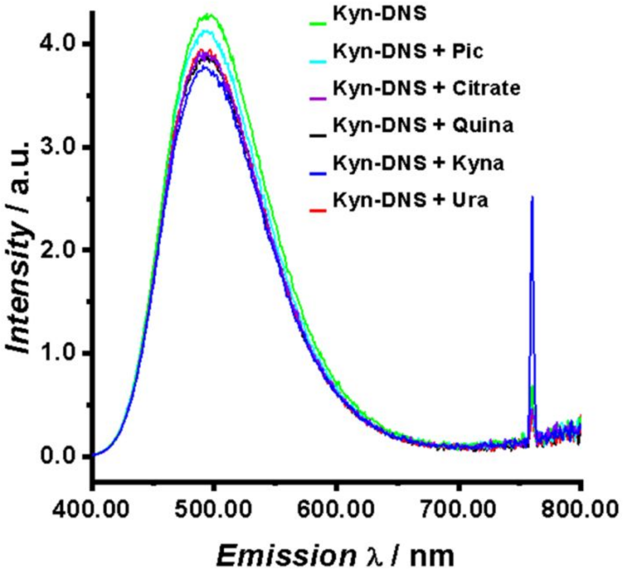

Figure S6. Fluorescence emission spectra of 3.00 µmol L^{-1} Kyn-DNS product with possible interferes in a 1:1 ratio. Addition of 10.00 µL for each possible interferent in 1.2 mmol L^{-1}, for a final volume of 4.00 mL, from the derivatization reaction.

Table S1. RGB data acquisition by Imagej software compared with the Color Grab app.

Channel	Image J ± SD	Color Grab ± SD	t-test	F-test
Red	185.00 ± 1.41	187.00 ± 2.08	1.17	1.48
	246.00 ± 1.40	253.00 ± 1.53	2.42	1.09
Blue	204.00 ± 1.31	202.00 ± 1.73	1.26	1.32
Vector	269.00 ± 2.36	374.00 ± 1.96	1.68	1.20

Table S2. RGB data, I-I_0 (a.u.) for the analytical curves using the Kyn-DNS/3Dchamber/FDIB method.

Kyn-DNS / µmol L^{-1}	Red ± SD	Green ± SD	Blue ± SD	Vector ± SD
0.50	18.19 ± 2.75	28.99 ± 2.72	20.57 ± 1.07	39.93 ± 2.38
1.00	29.70 ± 0.89	44.38 ± 1.03	25.80 ± 0.67	59.31 ± 0.78
3.00	58.09 ± 2.59	87.10 ± 0.84	34.69 ± 0.78	110.29 ± 1.30
5.00	85.96 ± 0.92	122.74 ± 0.73	47.23 ± 0.85	157.11 ± 0.66
7.00	121.98 ± 2.02	153.26 ± 2.82	58.77 ± 1.20	204.51 ± 1.31

Table S3. Relative error for the repeatability test inter-day for Kyn-DNS marker. RGB data, *I-I₀* (a.u.)

***[Kyn]* / µmol L^{-1}**	**Channel**	**Week 1/ a.u.**	**Week 2/ a.u.**	**Week 3/ a.u.**	**Average/ a.u.**	**RSD**
**0.50**	Red	25.00	27.00	28.00	26.67 (± 1.53)	5.73%
	Green	37.00	36.00	37.00	36.67 (± 0.58)	1.57%
	Blue	50.00	49.00	51.00	50.00 (± 1.00)	2.00%
	Vector	67.00	67.00	69.00	67.67 (± 1.15)	1.71%
**3.00**	Red	66.00	67.00	65.00	66.00 (± 1.00)	1.52%
	Green	104.00	101.00	98.00	101.00 (± 3.00)	2.97%
	Blue	90.00	95.00	89.00	91.33 (± 3.21)	3.52%
	Vector	152.00	154.00	147.00	151.00 (± 3.61)	2.39%
**7.00**	Red	124.00	108.00	118.00	116.67 (± 8.08)	6.93%
	Green	180.00	162.00	171.00	171.00 (± 9.00)	5.26%
	Blue	148.00	158.00	151.00	152.33 (± 5.13)	3.37%
	Vector	264.00	251.00	257.00	257.33 (± 6.51)	2.53%

Table S4. Recovery assay of the FDIB method compared to the HPLC UV technique.

**Sample**	**Added/ µmol L^{-1}**	**FDIB: Found ± SD (Recovery %)**	**HPLC-UV: Found ± SD (Recovery %)**	t-test	F-test
S1	0.50	0.50±0.03 (100%)	0.53±0.05 (106%)	0.89	1.12
	1.00	1.00±0.03 (100%)	1.10 ±0.08 (110%)	2.03	1.21
S2	0.50	0.46±0.03 (92.1%)	0.55±0.04 (110%)	2.42	1.32
	1.00	1.10±0.04 (106%)	0.94±0.06 (94.0%)	3.46	1.21
S3	0.50	0.46±0.05 (92.6%)	0.60±0.07 (120%)	2.81	1.70
	1.00	0.97±0.04 (97.4%)	0.90±0.08 (90.0%)	3.67	1.49

Table S5. Principles used by the metric system of the GREENess analytical calculator.

**Principles**	**Criterion**
1	Sample handling
2	Minimum sample size and number
3	In situ analysis
4	Low-energy operations and reduced reagent count
5	Automated and miniaturized method
6	No derivatization
7	Low generation of analytical waste
8	Multi-analytical or multi-parameter method
9	Minimal energy use
10	Reagents obtained from renewable sources
11	No toxic reagents
12	Increased operator safety

Table S6. Principles employed by the GAPI metric system.

**Principles**	**Criterion**
	**Sample preparation**
1	Collection
2	Preservation
3	Transport
4	Storage
5	Type of method
6	Scale of extraction
7	Solvents/reagents
8	Additional treatments
	**Reagents and solvents**
9	Amount
10	Health hazard
11	Safety hazard
	**Instrumentation**
12	Energy
13	Occupational hazard
14	Waste
15	Waste treatment

Table S7. Principles employed by the BADI metric system.

Principles	Criterion
1	Type of analysis
2	Single or multiple element analysis
3	Analytical technique
4	Simultaneous sample preparation
5	Sample preparation
6	Samples per hour
7	Reagents and materials
8	Preconcentration
9	Degree of automation
10	Sample quantity
